# Severe Familial Exudative Vitreoretinopathy, Congenital Hearing Loss, and Developmental Delay in a Child With Biallelic Variants in *FZD4*

**DOI:** 10.1001/jamaophthalmol.2022.2914

**Published:** 2022-08-11

**Authors:** Sarah R. van der Ende, Benjamin S. Meyers, Jenina E. Capasso, Mario Sasongko, Yoshihiro Yonekawa, Matthew Pihlblad, Jennifer Huey, Emma C. Bedoukian, Ian D. Krantz, Michael H. Ngo, Christopher R. McMaster, Alex V. Levin, Johane M. Robitaille

**Affiliations:** 1 Department of Biochemistry and Molecular Biology, Dalhousie University, Halifax, Nova Scotia, Canada; 2 Sidney Kimmel Medical College, Thomas Jefferson University, Philadelphia, Pennsylvania; 3 Pediatric Ophthalmology and Ocular Genetics, Flaum Eye Institute, University of Rochester, Rochester, New York; 4 Pediatric Genetics, Golisano Children’s Hospital, University of Rochester, Rochester, New York; 5 Wills Eye Hospital, Philadelphia, Pennsylvania; 6 Pediatric Ophthalmology and Strabismus, UPMC Children's Hospital of Pittsburgh, Pennsylvania; 7 Laboratory of Medicine and Pathology, University of Washington Medical Center, Seattle; 8 Roberts Individualized Medical Genetics Center, Children’s Hospital of Philadelphia, Philadelphia, Pennsylvania; 9 Department of Pharmacology, Dalhousie University, Halifax, Nova Scotia, Canada; 10 Department of Ophthalmology & Visual Sciences, Dalhousie University, Halifax, Nova Scotia, Canada

## Abstract

**Importance:**

Familial exudative vitreoretinopathy (FEVR) is a nonsyndromic autosomal dominant retinal disorder commonly caused by variants in the *FZD4* gene. This study investigates the potential role beyond ocular abnormalities for *FZD4* gene variants in patients with FEVR.

**Objective:**

To evaluate the role of *FZD4* in symptoms beyond those associated with FEVR through a patient with biallelic variants in *FZD4*.

**Design, Setting, and Participants:**

This case series included the DNA testing and phenotyping of 1 patient proband and her parents, combined with signaling assays, to determine the association of patient-derived compound heterozygous variants on FZD4 signaling and biologic function.

**Main Outcomes and Measures:**

*FZD4* genes were tested using next-generation sequencing and Sanger sequencing. Cell-based assays measured the effect of the variants on FZD4 signaling.

**Results:**

The proband presented with absent red reflexes from complete tractional retinal detachments diagnosed at 3 days of age and failed the newborn screening hearing test. Auditory brainstem response at 6 months of age showed bilateral mild to moderate high-frequency sensorineural hearing loss. The patient manifested developmental delays in speech and walking. Intravenous fluorescein angiography (IVFA) of the patient’s parents detected stage 1 FEVR. Genetic testing revealed 2 *FZD4* variants in the patient, each variant found in 1 parent. Signaling assays confirmed that the presence of both variants was associated with significantly worse signaling activity compared with the heterozygous state.

**Conclusions and Relevance:**

Results of this case series suggest that extraocular syndromic FEVR was associated with *FZD4* variants. The decrease in FZD4 signaling owing to the biallelic nature of the disease resulted in hearing deficits, developmental delays, and a more severe retinal phenotype.

## Key Points

**Question:**

Are *FZD4* variants associated with familial exudative vitreoretinopathy (FEVR) with extraocular features?

**Findings:**

This case series included a patient with biallelic *FZD4* variants with severe FEVR in infancy, congenital hearing loss, and developmental delay. Each parent was carrying 1 of the alleles and manifested mild FEVR; cell-based *FZD4* receptor-activation assays determined that *FZD4* function was dramatically decreased in the presence of these compound heterozygous variants.

**Meaning:**

Biallelic variants in *FZD4* can result in a severe ocular phenotype with systemic features, which may represent a novel syndrome.

## Teaser

This case series investigates if variants in the *FZD4* gene are associated with familial exudative vitreoretinopathy (FEVR) with extraocular features.

## Introduction

Familial exudative vitreoretinopathy (FEVR) is a heritable developmental disorder that prevents full retinal vascularization to the periphery. Depending on the extent of peripheral retinal nonperfusion, anomalous fibrovascular proliferation and exudation can occur causing retinal traction and retinal detachment in the childhood years.^1^

**Figure 1.**
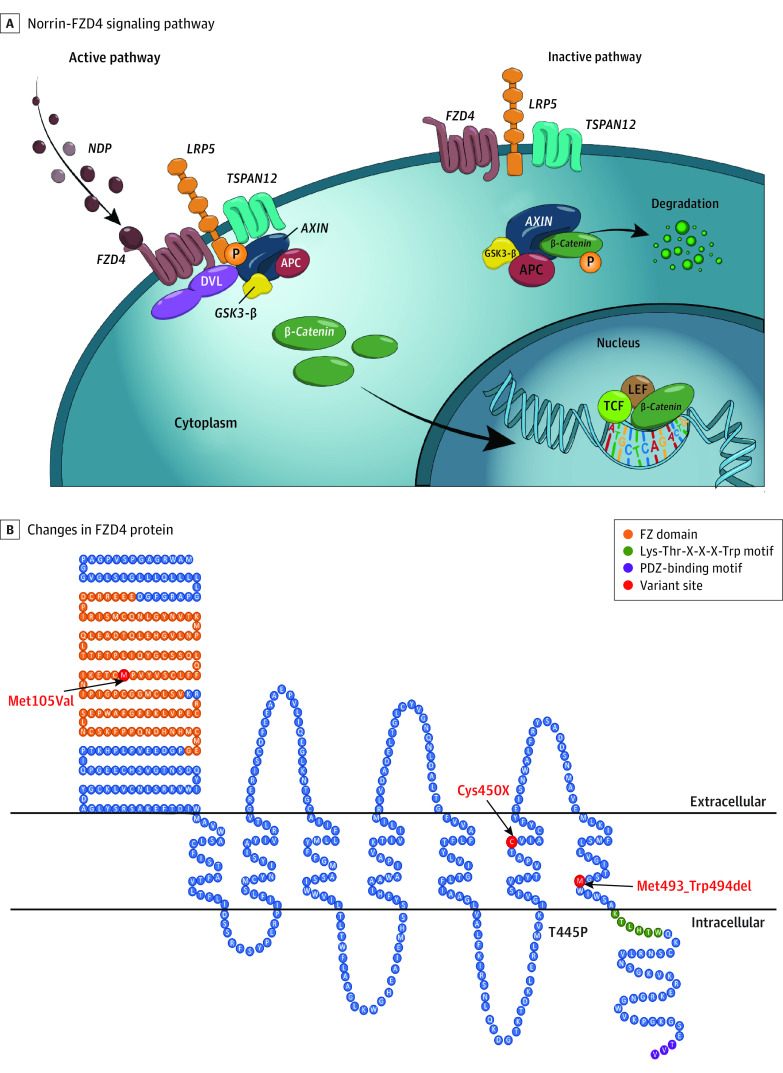
Rare Variants in the Norrin-FZD4 Signaling Pathway and Familial Exudative Vitreoretinopathy (FEVR) With Extraocular Phenotypes A, The Norrin-FZD4 signaling pathway. In the active form, the binding of Norrin (NDP) to FZD4, as part of the receptor complex with low-density lipoprotein receptor–related protein 5 (LRP5) and tetraspanin 12 (TSPAN12), results in the release of β-catenin and its translocation into the nucleus to induce T-cell factor (TCF)/lymphoid-enhancing factor (LEF)–mediated gene expression. In the absence of Norrin, β-catenin is phosphorylated (P) of by the axin-glycogen synthase kinase 3 (GSK3)–adenomatous polyposis coli (APC) destruction complex resulting in β-catenin–P degradation. B, The change in the FZD4 protein for each *FZD4* allele presented in this study. FZD4 p.Met105Val is located within the frizzled (FZ) cysteine-rich domain, a critical binding domain in the interaction with NDP. p.Cys450X results in the loss of the last 87 amino acids, including the entire intracellular tail, that contains a critical KTxxxW disheveled (DVL) binding motif. The control used in this study, p.Met493_Trp494del, results in the loss of 2 residues flush with the intracellular membrane surface. The PDZ-domain binds the intracellular proteins to mediate intracellular signaling (PDZ combines the first letters of the first 3 proteins discovered to share the domain: postsynaptic density protein, *Drosophila* disc large tumor suppressor, and zonula occludens 1 protein).

The majority of molecularly confirmed cases result in defects in components of the Norrin-FZD4 pathway^2^ (Figure 1A). Since first described in 2002,^3^ numerous variants in the *FZD4* gene have been reported to cause autosomal dominant FEVR with variable intrafamilial and interfamilial expressivity.^4,5^ Within the FZD4 signaling pathway, rare variants in the *NDP* gene result in Norrie disease (ND).^2^ The *NDP* gene encodes Norrin, the ligand for the FZD4 receptor. ND extraocular phenotypes can include sensorineural deafness, seizures, behavioral disorders, and developmental delay.^6,7^ To our knowledge, no case of FEVR with extraocular manifestations caused by *FZD4* has been reported.

We report a patient with severe FEVR, congenital hearing loss, and developmental delay. Genetic analysis revealed heterozygous biallelic variants in *FZD4* (Figure 1B**)**. We investigated the 2 variants identified in the patient for their individual and combined effect on Norrin-FZD4 signaling to further our understanding of *FZD4* variant genotypes with phenotype.

## Methods

Diagnostic evaluations, including examinations and genetic testing, were performed in a clinical setting. Our laboratory was regulated by the research ethics boards at both the IWK Health Centre and Dalhousie University. Clinical genetic testing used the Vitreoretinopathy SmartPanel, version 1 (1615 primer sets) performed by polymerase chain reaction and next-generation sequencing (Molecular Vision Laboratory). Variants were confirmed by Sanger sequencing. Coding regions and immediately flanking exon-intron splice sites were examined. Testing included 19 genes (*FZD4*, *NDP, LRP5, COL2A1, COL4A3, COL4A4, COL4A5, COL9A1, COL9A2, COL11A1, COL11A2, COL18A1, KCNJ13, VCAN, TSPAN12, CAPN5, ZNF408, KIF11, ATOH7*). Further genetic testing included a single-nucleotide variation array, karyotype, and AUDIOME genetic testing (Children’s Hospital of Philadelphia), an exome-sequencing–based hearing loss panel that includes deletion and duplication analysis of 122 genes.^8^

Details of the methods for determining Norrin-FZD4 signaling are described in detail in the eMethods of the Supplement. Briefly, we compared Norrin-FZD4 signaling of each of the *FZD4* patient variants in the homozygous and heterozygous states. This case series followed the CARE guidelines for case reports.

## Results

A girl aged 2 years and 8 months presented for evaluation of FEVR. She was born full term after an uncomplicated pregnancy. After failing the newborn screen, auditory brainstem response at 6 months old showed congenital bilateral mild to moderate high-frequency sensorineural hearing loss. Her parents observed that she had visual problems. At 3 days old, she was found to have an absent right red reflex and was diagnosed with bilateral complete tractional retinal detachments. At 2 years of age, despite surgery in each eye, she had light perception OD and no light perception OS. Examination under anesthesia showed total retinal detachments that apposed the residual posterior capsule in both aphakic eyes. The patient was also noted to have significant developmental delays in speech and walking.

**Figure 2.**
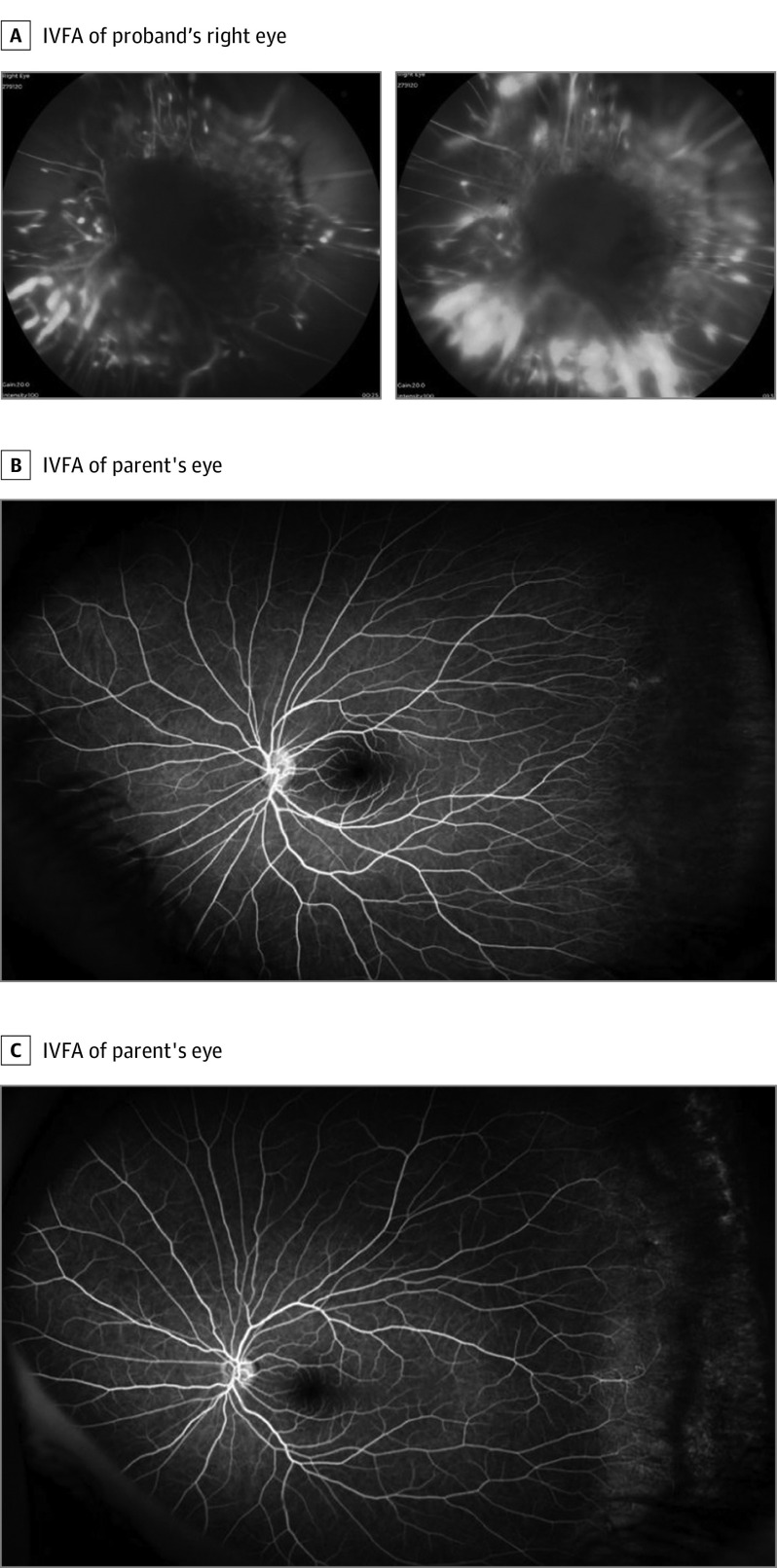
Presentation of Familial Exudative Vitreoretinopathy (FEVR) in the Patient Proband and Parents A, Intravenous fluorescein angiography (IVFA) of the proband’s right eye. Left panel, there is an open-funnel detachment with peripheral nonperfusion, vessel pruning, and abnormal vascular patterning. A later-phase IVFA (right panel) of the proband’s right eye shows severe leakage of peripheral vessels. In both parents (B) and (C), FEVR was characterized by supernumerary vessels, peripheral avascularity, and vascular pruning and branching. B, IVFA of patient’s parent with the p.Cys450X variant showing temporal retina nonperfusion only in the left eye. C, IVFA of patient’s parent with the p.Met105Val variant showing peripheral nonperfusion and leakage from distal vessels without evidence of neovascularization.

Intravenous fluorescein angiography (IVFA) of the patient and parents was performed. The patient showed bilateral open-funnel detachments with peripheral avascular retina (Figure 2A), staining of the terminal vessels, and diffusely anomalous vasculature. Both parents manifested stage 1 FEVR (Figure 2B and C). Another relative had myopia and bilateral retinal detachments but was not available for further examination.

Genetic testing revealed 2 heterozygous *FZD4* variants in the patient, each variant found in 1 parent (Figure 1B). The *FZD4* missense variant in exon 2, c.313A>G (p.Met105Val), inherited from the father, is located in a key domain that interacts with Norrin and was previously reported and shown to reduce Norrin-FZD4 signaling.^5,9^ The nonsense *FZD4* variant in exon 2, c.1350T>A (p.Cys450X), inherited from the mother, truncates the protein in the middle of the sixth transmembrane domain and completely lacks the intracellular tail that is key to transmitting the Norrin signal intracellularly.^10^ Karyotyping and single-nucleotide variation array were normal. AUDIOME testing revealed a heterozygous variant in the *PTPRQ* gene, p.Arg1931Gln:c.5792G>A (NM_001145026). Rare variants in the *PTPRQ* gene are known to cause deafness in the homozygous recessive or heterozygous dominant state. The known dominant allele (OMIM 603317) that causes hearing loss is a stop codon near the C terminus. The arginine to glutamine change reported in this patient is for a very similar amino acid as far as shape and charge, and prediction algorithms Sorting Intolerant From Tolerant (SIFT)^11^ and Protein Variation Effect Analyzer (PROVEAN)^12^ predict a benign change. It is highly unlikely that the missense pathogenic variant observed in *PTPRQ* was the cause of hearing loss.

**Figure 3.**
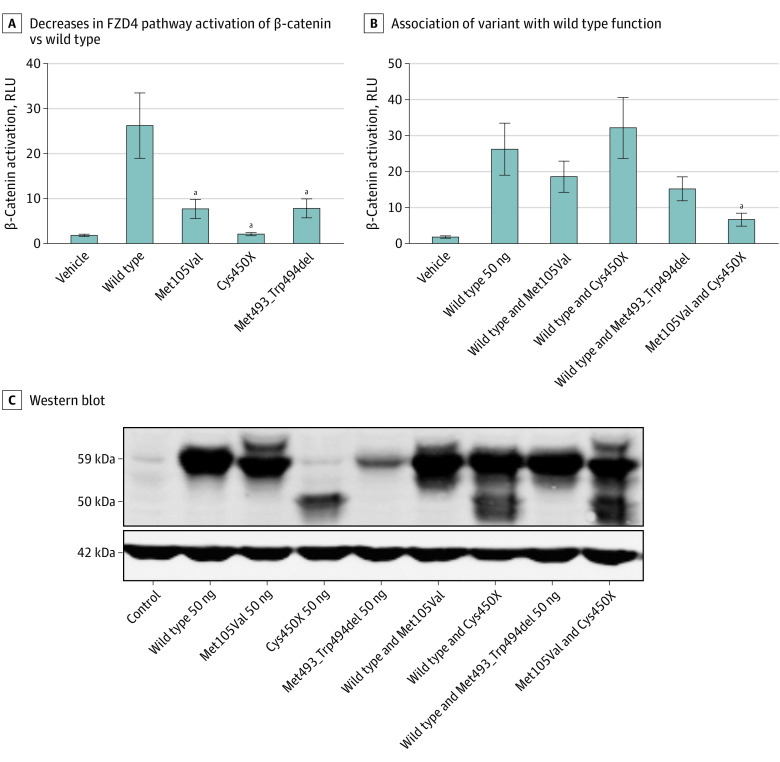
Effect of the *FZD4* Patient-Derived Variants on Norrin-FZD4 Signaling Different species of FZD4 were produced using transfection of variants in HEK293T cells, along with LRP5 and TSPAN12, with pathway activation measured after adding Norrin. A, All 3 variants display significant decreases in FZD4 pathway activation of β-catenin compared with wild type. The p.Met105Val and p.Met493_Trp494del variants show a marked decrease in functioning, whereas the p.Cys450X truncated variant fails to activate the pathway entirely. B, To determine the effects of the variant on wild type function, 2 different FZD4 species were simultaneously used in the FZD4 signaling assay. When the p.Met105Val and p.Cys450X FZD4 variants were expressed together, there was a significant 4-fold decrease in FZD4 signaling. Expression of the parental p.Met105Val or p.Cys450X variant with wild type FZD4, as well as the known p.Met493_Trp494del dominant allele, did not significantly affect FZD4 signaling. As inheriting 1 *FZD4* variant in humans is known to cause FEVR with variable expressivity, the cell-based assay appears not to be sensitive enough to accurately predict even known *FZD4* individual variant effects on disease severity when expressed in trans with wild type *FZD4*. C, Western blot assessing protein expression of each FZD4. The band corresponding to the p.Cys450X variant appears lower at 50 kDa as opposed to 59 kDa in wild type FZD4, owing to the introduction of the premature stop codon that reduces the protein size by 87 residues. The bands demonstrate that all proteins were successfully produced in each cell experiment thus validating the signaling assays results.

We used a well-established assay for the Norrin-FZD4 pathway to determine the effect of each variant in a homozygous and compound heterozygous state. As a control, we used a previously established dominant *FZD4* allele, FZD4 p.Met493_Trp494 del.^3^ For this assay, baseline activity was the absence of Norrin in the assay. When expressed singularly, the p.Met105Val and p.Met493_Trp494del variants resulted in a 50% decrease in Norrin-FZD4 signaling, whereas the p.C450X variant displayed a complete loss of FZD4 signaling (Figure 3A). We next compared the outcome of the variants when present along with wild type FZD4 to mimic the genotype observed in each parent, as well as the p.Met105Val variant together with p.C450X to mimic the genotype of the affected offspring. The wild type–p.Met105Val combination resulted in a small but insignificant decrease in signaling, whereas the wild type–p.C450X combination did not affect FZD4 signaling. Importantly, when expressed together, the p.Met105Val–p.C450X combination resulted in a level of Norrin-FZD4 signaling 4-fold lower than wild type (Figure 3B). As expected, the known dominant allele p.Met493_Trp494del decreased Norrin-FZD4 by almost 50%. We assessed expression of each allele by Western blot and confirmed that all alleles resulted in the production of FZD4 protein (Figure 3C).

## Discussion

This case series identified a patient with biallelic *FZD4* variants, severe FEVR, and an extraocular phenotype, including sensorineural hearing loss and developmental delay, potentially associated with a new syndrome. Biochemical characterization of patient-derived *FZD4* variants in a heterozygous state with each other and with a wild type *FZD4* allele determined that Norrin-FZD4 signaling was substantially reduced in the state mimicking the biallelic offspring.

ND is an inherited disorder that typically presents in infancy with a severe FEVR phenotype accompanied by progressive hearing loss and developmental delay in some patients.^13,14^ ND is attributable to rare variants in the *NDP* gene, which encodes Norrin, the ligand for the FZD4 receptor.^9^ This patient presented with features similar to those seen in ND. One explanation is that the *NDP* variants that cause ND, and the biallelic *FZD4* variants present in this patient, result in a dramatic loss of Norrin-FZD4 signaling, causing the extraocular phenotypes. Consistent with near-complete loss of Norrin-FZD4 signaling observed in our proband, *Fzd4*^−/−^ knockout mice display congenital retinal hypovascularization similar to that observed in FEVR, as well as progressive inner-ear vascular atrophy resulting in hearing loss and progressive cerebellar degeneration.^9^ Alternatively, formally testing the parents for hearing loss and a broader genetic search beyond the AUDIOME panel may reveal a separate etiology for the patient’s auditory deficit. Also, it was not possible to determine the contribution of the combined early-onset severe visual and mild to moderate hearing loss to the developmental delays. The almost complete overlap in the patients’ phenotype with ND, coupled with the same phenotype observed in *Fzd4* knockout mice, may imply that severe defects in Norrin-FZD4 signaling were associated with the extraocular phenotypes observed, and hearing testing should be performed in patients with biallelic *FZD4* variants.

## Conclusions

In this case series investigation, a syndrome was presented that was associated with biallelic variants in the *FZD4* gene and early-onset severe FEVR accompanied by hearing loss and developmental delay. A very severe defect in Norrin-FZD4 signaling may be the mechanism by which the extraocular phenotypes may manifest.

## Notes

Supplement.
eMethods
