# Supplementary material for: Severe Familial Exudative Vitreoretinopathy, Congenital Hearing Loss, and Developmental Delay in a Child With Biallelic Variants in FZD4
Source: JAMA Ophthalmol. 2022 Aug 11;140(9):889–93. doi: 10.1001/jamaophthalmol.2022.2914 (PMC9372905; doi:10.1001/jamaophthalmol.2022.2914)
Supplement: Supplement. — eMethods [file jamaophthalmol-e222914-s001.pdf]

Supplementary Online Content

van der Ende SR, Meyers BS, Capasso JE, et al. Severe familial exudative vitreoretinopathy, congenital hearing loss, and developmental delay in a child with biallelic variants in *FZD4*. *JAMA Ophthalmol*. Published online August 11, 2022. doi:10.1001/jamaophthalmol.2022.2914

eMethods

This supplementary material has been provided by the authors to give readers additional information about their work.

© 2022 van der Ende SR et al. *JAMA Ophthalmology.*

eMethods

Plasmid generation. Expression vectors for pRK5-*FZD4*, pRK5-*LRP5*, and pRK5-*Renilla* luciferase were a gift from Dr Jeremy Nathans. *TSPAN12*-ddk was purchased from Origene (RC206943). The following plasmids were purchased from Addgene: M50 Super 8x TOPFlash (Plasmid#12456), c-Flag pcDNA3 (Plasmid#20011).

Site-directed mutagenesis to create the patient-derived FZD alleles was performed using the Quikchange II kit (Agilent) following the manufacturers protocol. Primers were designed and purchased from IDT and were:

FZD4 p.Met105Val FWD: 5' GTTCTGTTTGTGTGCCAATGTGCAC 3'

FZD4 p.Met105Val REV: 5' CACATTGGCACACAAACAGCCCAAAGG 3'

FZD4 p.Cys450X FWD: 5' GTTCCTGCAACGTGAATTGCCTG 3'

FZD4 p.Cys450X REV: 5' GCAATCACTCACGTTGCAGGAACTGTG 3'

FZD4 p.Met493_Trp494del FWD: 5' GCATCACTTCAGGCTTTGGTGTGCCAAAACTC 3'

FZD4 p.Met493_Trp494del REV: 5' GGCAGACCAAATGCCTGAAGTGATGCCCACCAAC 3

Open reading frames were sequencing to confirm the presence of the patient-derived pathogenic variants and control *FZD4* variant as well as the absence of extraneous changes to the coding region.

Cell Culture and transfection the luciferase assays. HEK293T cells were maintained in Dulbecco's modified Eagle medium supplemented with 10% fetal bovine serum (Sigma). HEK293T cells were transfected at 100,000 cells per well in 24 well plates. Four hours later,

© 2022 van der Ende SR et al. *JAMA Ophthalmology.*

cells were transfected with 1.5 mL FUGENE6 transfection reagent with equal amounts of DNA. Ratios for the reporter plasmids were set up as 150 ng M50 reporter: 25 ng Renilla reporter. For investigating the individual effects of the *FZD4* variants, all wells received 100 ng LRP5, 50 ng TSPAN12, and 50 ng of a FZD4 variant. For investigating the compound effects, wells received 100 ng total of FZD4, 50 ng each of two species. Samples were stimulated with Norrin or a vehicle control 8-10 hours post-transfection. Samples were processed using the Dual Luciferase Assay kit (Promega) 24 hours after transfection. Firefly:Renilla luciferase ratios were determined, normalized to controls, and expressed as relative luciferase units (RLU). Statistical significance was determined using one way analysis of variance (ANOVA) and Student's T test at $p<0.05$.

Western blots. Anti-FZD4 antibody was Invitrogen (145901). Anti-actin C4 was purchased from Santa Cruz (sc-47778). IRDye^{r} 800CW Goat-anti-mouse IgG and IRDye^{r} 800CW Goat-anti-rat IgG secondary antibodies were bought from Li-Cor. Samples were set up and transfected as previously described. 24 hours post-transfection, samples were harvested using Laemmli buffer and incubated at 37 degrees for 20 minutes. The samples were run on a 10% poly-acrylamide gel and transferred to a nitrocellulose membrane. The membranes were probed using the antibodies listed above.

© 2022 van der Ende SR et al. *JAMA Ophthalmology.*
